# Clear Cell Hidradenoma in a Pregnant Female

**DOI:** 10.7759/cureus.27676

**Published:** 2022-08-04

**Authors:** Sweta Sahu, Ravishankar Ranganatha, Hari krishna Uppalapati, Resham Tanna, Christian Perryman, Umesh Batura, Rakshith G R, Kailas C T

**Affiliations:** 1 Surgery, Jagadguru Jayadeva Murugarajendra Medical College, Davanagere, IND; 2 Surgery, Karnataka Institute of Medical Sciences, Hubballi, IND; 3 Medicine, St. Martinus University Faculty of Medicine, Willemstad, CUW; 4 Surgery, Spartan Health Sciences University, Vieux Fort, LCA; 5 Surgery, Saint James School of Medicine, Grenadines, VCT; 6 Surgery, American University of Antigua, St. Johns, ATG

**Keywords:** gynecologic oncology, clear cell tumors, acrospiroma, sweat gland neoplasms, eccrine sweat glands, clear cell hidradenoma

## Abstract

Solid cystic hidradenoma, or clear cell hidradenoma, is a distinct and histologically rare tumor formed at the sweat glands, found mainly in adults and majorly among women. In this case, a 26-year-old female presented with asymptomatic swelling in her left inguinal area. Similar cases have been discussed in the literature considering the same kind of tumor. The present case is reported owing to the rarity of the type of tumor in terms of size and region of occurrence with the associated condition of pregnancy.

## Introduction

Hidradenoma is a rare kind of tumor originating in the sweat glands. Hidradenomas were previously thought to show just eccrine differentiation. However, now they are reported to show both apocrine differentiation and eccrine differentiation [1,2]. This type of tumor is common in adults, mainly among females. These lesions are very rare in children [3,4]. These tumors are firm dermal nodules, with sizes ranging from 5 to 30 mm. The lesions grow on the overlying epidermis, which can be either ulcerated or thick. Its growth is very slow, and a serous discharge is observed. These lesions are usually solitary. The most common anatomical sites for these tumors are on the face, scalp, proximal limbs, and anterior trunk [3]. Here, we discuss the case of a 26-year-old female who presented with a swelling and an asymptomatic mass in the left inguinal region.

## Case presentation

Clinical summary

**Figure 1:**
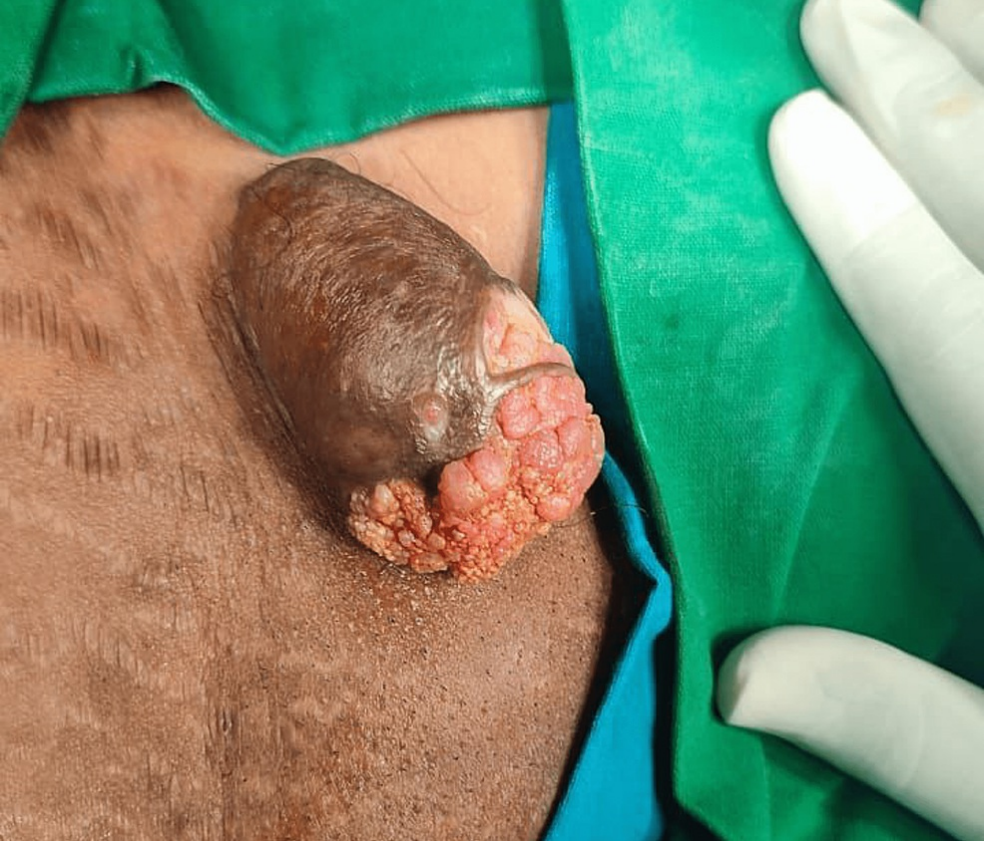
Swelling in the left inguinal region.

A 26-year-old female presented with an asymptomatic, solitary, 8 × 4 cm swelling in the left inguinal region. She was in her fourth month of the post-partum period. Her history revealed that during her first trimester she had noticed a small (about 1 × 1 cm) swelling in her left inguinal region. However, owing to the asymptomatic nature and the small size, no action or treatment was sought. However, during her third trimester, the size of the swelling increased to about 4 × 5 cm. The patient consulted her local clinician and was advised to consult the surgery clinic after her delivery. After the delivery, the patient chose not to consult because of the painless nature and no change in the size of the swelling. After a while, the swelling began to increase in size which caused her discomfort during her routine activities in everyday life. In her fourth month of post-partum, she finally presented to the Department of Surgery of our hospital with swelling of 8 × 4 cm (Figure 1).

**Figure 2:**
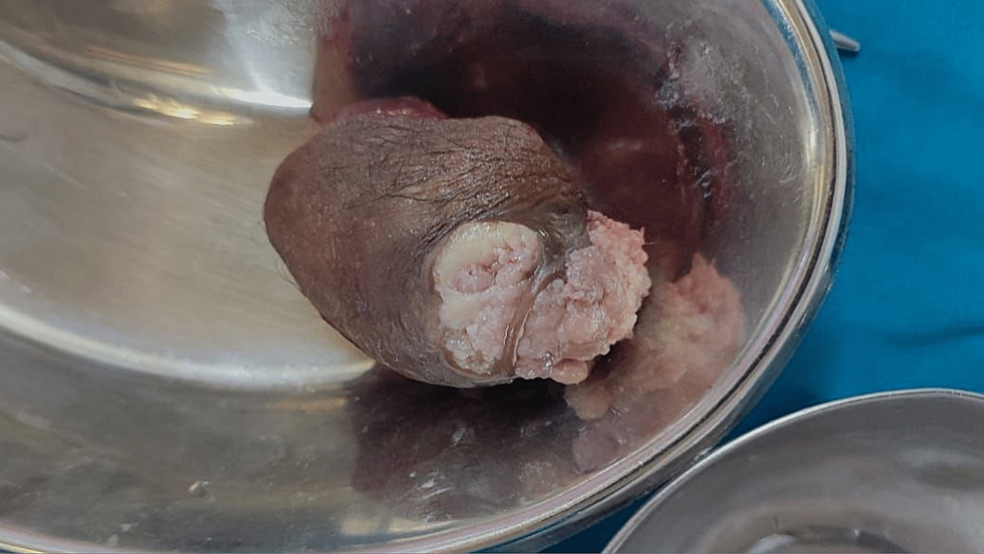
Excised lesion for sampling.

The lesion was irregular in shape with small ulcers and a small amount of discharge of serous fluid in the apex. The lesion was asymptomatic because it grew rapidly. Moreover, the tumor touched the petty coat (a type of garment) and undergarments and caused a little pain with serous discharge and discomfort. Ultrasonography revealed a rounded well-circumscribed nodule predominantly cystic with mural solid part as papillary projection. The lesion was situated at the dermis extending to the subcutaneous tissue. Doppler study showed that the lesion was highly vascular within the solid parts of the lesion and its periphery. Hence, it was diagnosed as a calcified hemangioma. As a part of the definitive diagnostic procedure, a wide local elliptical excision was performed. After the surgery, the lesion was sampled and sent to the pathology department (Figure 2).

Pathological findings

**Figure 3:**
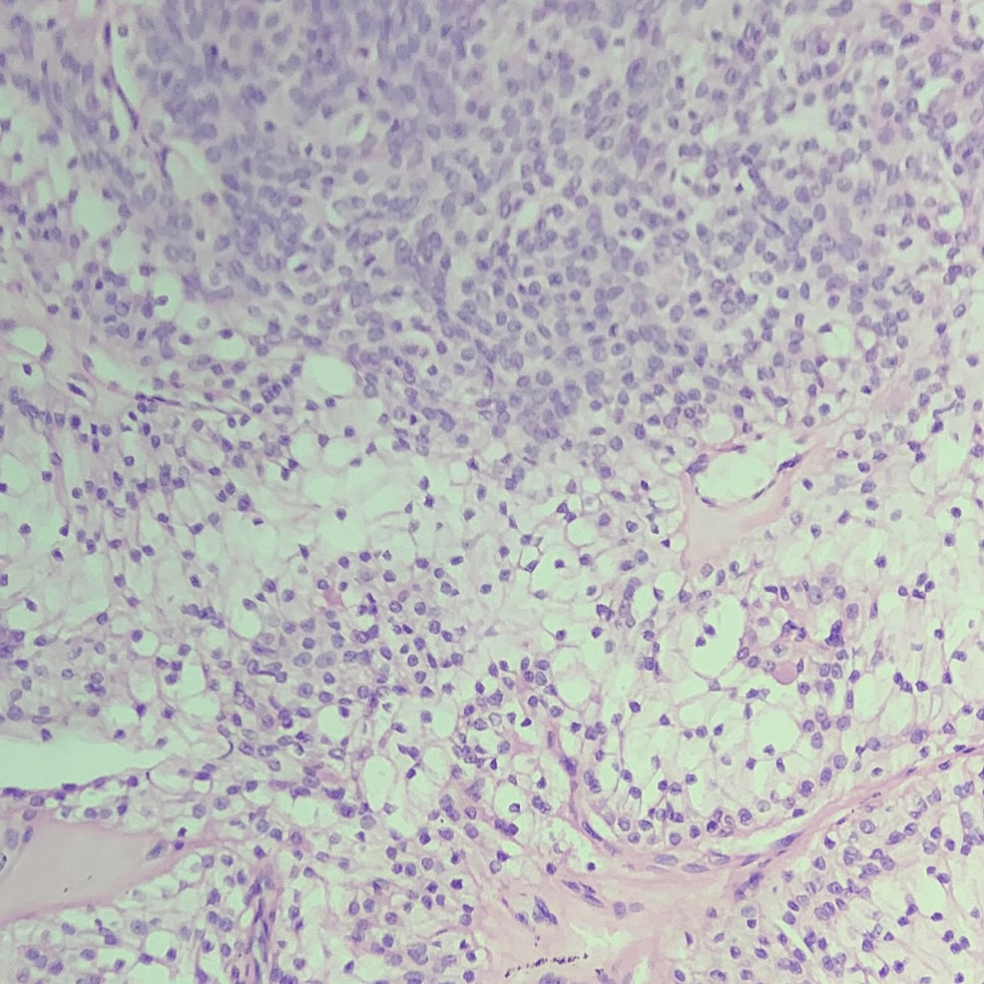
Hidradenoma with predominant clear cells and polyhedral cells.

The specimen used for macroscopic tests consisted of the skin with hair attached soft-tissue mass measuring 8 × 4 × 3 cm. The external surface showed the presence of ulcers. The cut area was friable, grayish white, and showed degenerative areas. The specimen underwent a histopathological analysis showing the same type of morphology. Multiple sections from the mass showed a dermal neoplastic lesion with focal attachment to the overlying epidermis. The neoplastic lesion was considered to comprise cystic and solid components. The solid components were in sheets, nests, and nodules and showed two kinds of cells, polyhedral cells had a vesicular nucleus and eosinophilic cytoplasm, and clear pale cells with a distinct cell membrane and eccentric nucleus (Figure 3). The cystic spaces were lined by cuboidal to columnar cells. The intervening stroma was hyalinized to sclerotic. Few nests showed stromal epithelial retraction.

**Figure 4:**
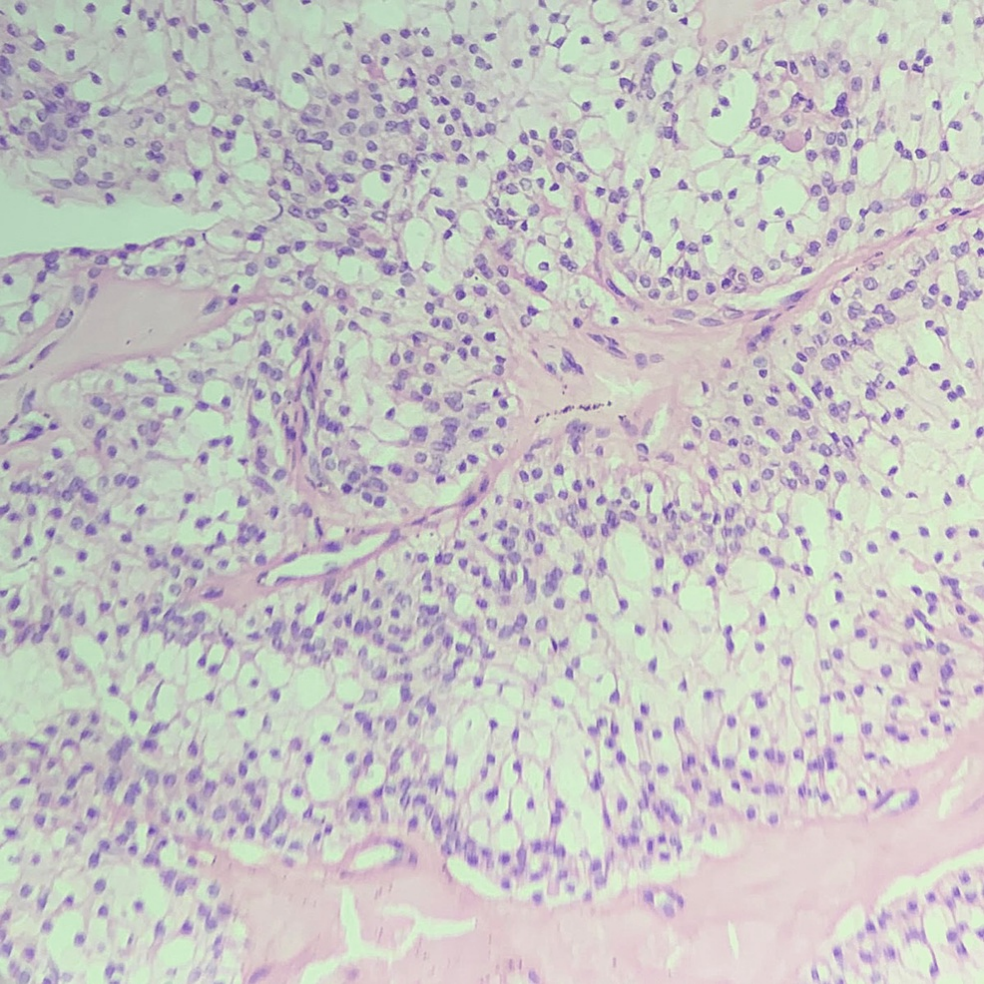
Solid cystic variant of hidradenoma with a bimodal cell population.

Minimal mitotic activity was noticed. The diagnosis of solid cystic hidradenoma/clear cell hidradenoma was confirmed (Figure 4).

## Discussion

**Figure 5:**
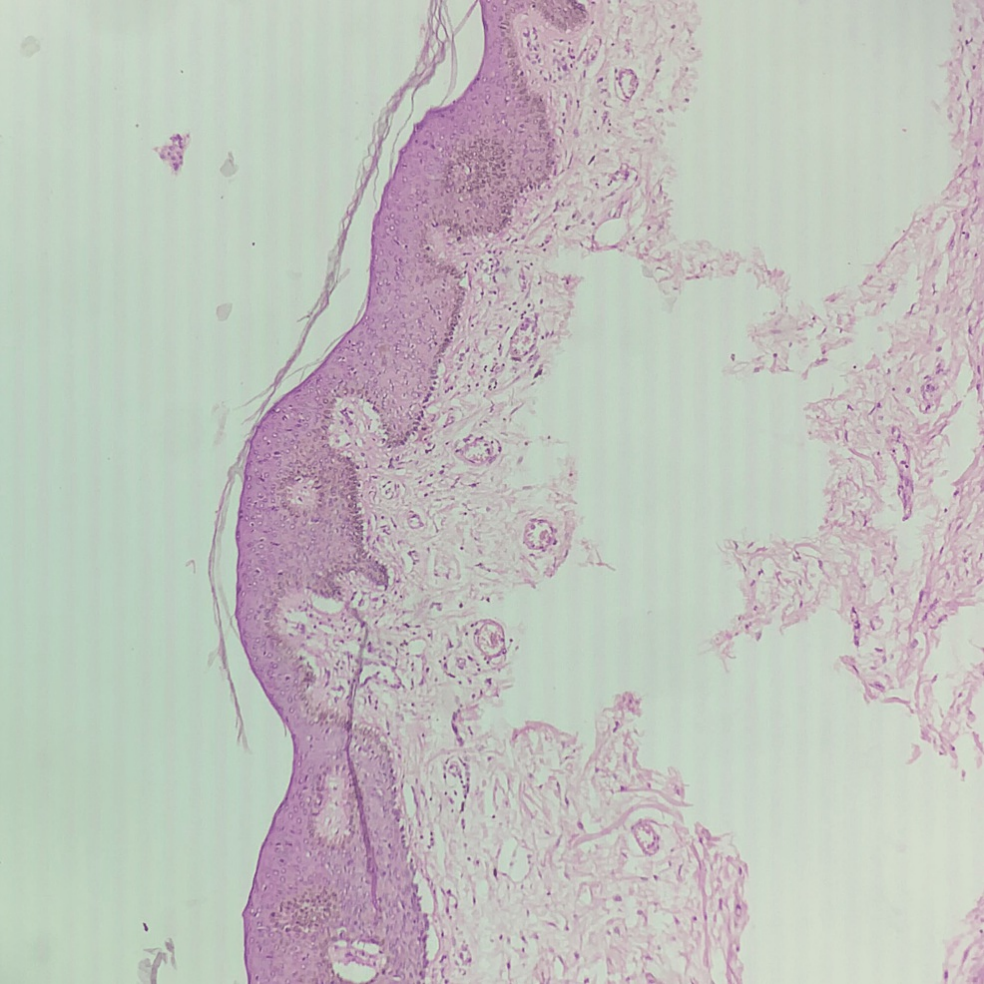
Microscopic view of the tumor with epidermis.

Liu described clear cell hidradenoma, also called eccrine acrospiroma of the skin, as the clear cell papillary carcinoma of the skin in 1949. Later, this was considered in different designations [5]. Acrospiromas, or nodulocystic hidradenoma, or clear cell hidradenoma, are rare histological variants of sweat gland origin. It was previously believed that these tumors just showed eccrine differentiation. However, now it has been considered that it can show either apocrine or eccrine differentiation [1,2]. These tumors occur among adults and most commonly in women compared to men. These lesions are usually rare among children [3,4]. The lesion presents as a gradually enlarging, solitary, firm, asymptomatic tumor, with a nodule size of 5-30 mm, and grows on the overlying epidermis. These tumors also show serous fluid discharge, whereas other tumors normally ulcerate. In this case, the rarest complaint was slight tenderness. Lesions can appear in any anatomical area [5]. The tumor’s histopathological appearance of clear cell hidradenoma is very typical. These can be grouped into two kinds of cells: clear and eosinophilic cells with a ductal cystic space lined with a low cuboidal cell [6]. Focal squamous differentiation is also observed [7]; however, it was not observed in our patient. In our case, there were no neoplastic cells in the epidermis while the dermis had a bimodal population of neoplastic cells (Figure 5).

For these types of tumors, malignant transformation is quite uncommon, seen in about 1% of cases according to the literature [8]. Clinical diagnosis can be confirmed in the cases where the tumor grows on the epidermis, specifically if any discharge is observed earlier. Ulcerated lesions can be similar to basal cell carcinoma. For clinical inspection, the non-diagnostic factors are the dermal nodules. Benign lesions can be cured by surgical excision. Local re-occurrence is uncommon. Malignant eccrine hidradenoma may metastasize [9,10]. Clear cell hidradenomas are different from concordant lesions such as trichilemmoma, hemangioma, follicular cyst, glomus tumor, leiomyoma, cutaneous lymphoma, dermatofibrosarcoma protuberans, or adnexal tumors or adjunctive sweat gland which are indistinguishable clinically [11,12]. A comprehensive surgical excision is important to manage clear cell hidradenoma as nodules have a higher potential for tumor recurrence. For surgical excision, a wide perimeter of the tumor is mandatory to get a detailed histological confirmation for ensuring minimal probable re-occurrence and evaluating any further future malignant transformation. The neoplasm can be surgically removed with a broad perimeter usually by preferred therapy. Malignant transformation is very rare; however, neoplasms such as clear cell hidradenocarcinoma recur. Hence, there are no plans for any repeated surgery. We are reporting this case for its rarity in size and occurrence in this region with the associated condition of pregnancy.

## Conclusions

Solid cystic hidradenoma, or clear cell hidradenoma, is a distinctive and rare tumor usually originating in the sweat glands. It is common in adults, and especially among women. In our case, histopathology showed a positive margin in the section. However, because the tumor was benign and slow growing, the progress of the tumor and the patient’s condition could be tracked with regular follow-ups. Hence, there are no plans for any repeated surgery. We are reporting this case because of its rarity in size and region and the associated condition.
